# Fabrication and Characterization of Scaffolds of Poly(*ε*-caprolactone)/Biosilicate® Biocomposites Prepared by Generative Manufacturing Process

**DOI:** 10.1155/2019/2131467

**Published:** 2019-02-03

**Authors:** Daniel Aparecido Lopes Vieira da Cunha, Paulo Inforçatti Neto, Kelli Cristina Micocci, Caroline Faria Bellani, Heloisa Sobreiro Selistre-de-Araujo, Zilda Castro Silveira, Marcia Cristina Branciforti

**Affiliations:** ^1^Department of Materials Engineering, University of Sao Paulo, Sao Carlos, SP, Brazil; ^2^Three-Dimensional Technologies Division, Renato Archer Information Technology Center, Campinas, SP, Brazil; ^3^Department of Physiological Sciences, Federal University of Sao Carlos, Sao Carlos, SP, Brazil; ^4^Department of Mechanical Engineering, University of Sao Paulo, Sao Carlos, SP, Brazil

## Abstract

Scaffolds of poly(*ε*-caprolactone) (PCL) and their biocomposites with 0, 1, 3, and 5 wt.% Biosilicate® were fabricated by the generative manufacturing process coupled with a vertical miniscrew extrusion head to application for restoration of bone tissue. Their morphological characterization indicated the designed 0°/90° architecture range of pore sizes and their interconnectivity is feasible for tissue engineering applications. Mechanical compression tests revealed an up to 57% increase in the stiffness of the scaffold structures with the addition of 1 to 5 wt.% Biosilicate® to the biocomposite. No toxicity was detected in the scaffolds tested by* in vitro* cell viability with MC3T3-E1 preosteoblast cell line. The results highlighted the potential application of scaffolds fabricated with poly(*ε*-caprolactone)/Biosilicate® to tissue engineering.

## 1. Introduction 

Tissue engineering is a multidisciplinary area of emerging research that applies the concepts of biological sciences and engineering for the development and manipulation of cells, tissues, or organs to restore, maintain, or support the function of damaged tissues [1]. Scaffolds are biological substitutes that act as a transient extracellular matrix (ECM) composed of a porous three-dimensional structure that supports the growth and restoration of tissues [2, 3]. In-depth studies on the choice of a biomaterial or biocomposite that constitutes the scaffolds are fundamental for the obtaining of structures that perform different functions in the regeneration of tissues [4]. Properties, such as biocompatibility, biodegradation, and bioactivation, specifically related to structure morphology and mechanical properties are essential for the selection of biomaterials to be applied in tissue engineering.

Poly(*ε*-caprolactone) (PCL) is an aliphatic polyester of excellent biocompatibility, biodegradability, and mechanical strength commonly addressed in several studies on tissue engineering [5, 6]. The first studies of synthesis of PCL were developed in the early 20th century by researchers interested in the production and understanding of the use of synthetic polymers that might be degraded by the body [7]. PCL-related research was intensified only after the emergence of the tissue engineering concept and the consequent demand for use of biopolymers in the manufacture of scaffolds [8]. PCL has low melting temperature of approximately 60°C, relative high degradation temperature (around 300°C), and hydrophobic character, which make it a highly suitable biopolymer for the production of scaffolds by additive manufacture [5, 8]. However, PCL exhibits low bioactivity, which restricts its use as biomaterial for tissue regeneration in clinical applications. In order to overcome this limitation, studies have demonstrated the incorporation of osteoconductive and/or osteoinductive bioactive ceramic phases, such as hydroxyapatite, bioactive glasses, or glass-ceramics, into a PCL matrix increases its bioactivity and improves the mechanical properties of the polymer [9–13].

Biosilicate®, a particulate fully crystalline bioactive glass-ceramic, has proven a versatile, multipurpose biomaterial to be applied in bone tissue engineering and an efficient alternative for the treatment of dentin hypersensitivity [14–16]. It is obtained by special nucleation and growth thermal treatments of the quaternary Na_2_O–CaO–SiO_2_–P_2_O_5_ system [17]. The resulting fully crystallized bioactive glass-ceramic exhibits improved mechanical properties over glasses and high bioactivity, and, after milling, the particles are less sharp and abrasive, which is an important feature for their use in different manufacturing processes. Peitl et al. [18] demonstrated a controlled crystallization of glasses of the same system increased their average bending strength from 75 MPa of the parent glass to 210 MPa.* In vitro*,* in vivo,* and clinical studies [14–16, 19–25] have shown the efficiency of Biosilicate® for regenerating bone tissue due to its features, such as high bioactivity, osteoconductivity, osteoinductivity, osteoconductivity, noncytotoxicity, nongenotoxicity, and antibacterial properties. Such excellent biological and special mechanical properties have made Biosilicate® promising bioceramic reinforcement for the production of scaffolds.

Techniques for a three-dimensional fabrication of scaffolds can be classified as direct or indirect. Indirect techniques use a mold for generating pores in the production of scaffolds, whereas, in direct techniques, pores are intrinsically constructed with the three-dimensional model by the additive manufacturing process [26]. Additive manufacturing is a fabrication mode appropriate for scaffolds. Among its advantages are accuracy of material deposition and mainly reproducibility, which enables the generation of homogeneous structures of controllable porous morphology within specifications for applications in tissue engineering [27, 28].

An experimental Fab@CTI 3D printer (middle-end 3D printer) was used for the fabrication of scaffolds. Inspired in the Fab@Home project, it offers open structure (mechanical design, interface program, and control system), process control, and use of low amounts of material for processing [29]. Fab@CTI 3D printer is composed of four subsystems, namely, drive system (base), mechanism for material deposition (based on a single screw), interface program, and monitoring system. The base-related drive system has a Cartesian platform controlled by three stepper motors coupled to movement elements (lead screws). Displacements in X and Y directions occur in a fixed plan, while the platform moves in the Z direction. The deposition mechanism is composed of a driven system and a subsystem formed by mechanical coupling, miniscrew/barrel, silo, and nozzle that perform transportation, compression, flow control, and deposition of the raw material for the generation of the final three-dimensional structure or part of it [29, 30]. The mechanism for material deposition or, simply, “deposition head,” accepts a 250°C maximum processing temperature and medium torque around 26 Nm. Such design and operational parameters were estimated to process polymer and polymer composites.

The scaffolds were geometrically generated by BioScaffold PG software, which enables different configurations related to deposition directions. In the present study, the 0°/90° architecture was established as the best configuration for the control of the scaffold pore size. Its parameters were set to the layer height, spacing between the extruded filaments, number of spaces, number of layers, and diameter of the desired extruded filament. They were compacted and exported to a file extension compatible with the operating/control system of the 3D printer, e.g. STL, G-Code.txt, or Points.txt [31]. The software monitors the entire manufacturing process of the scaffold and provides all processing information, such as expected time for production, data on a single screw-based head, and rotation speed of the X, Y coordinates [29, 30].

Domingos et al. [27] investigated the influence of nano- and microhydroxyapatite particles on the in vitro biological and mechanical performance of PCL/hydroxyapatite scaffolds. The three-dimensional scaffolds were produced using an extrusion-based additive manufacturing system. The results from compression tests evidenced that PCL/microhydroxyapatite scaffolds presented a higher value of compressive modulus than PCL/nanohydroxyapatite scaffolds. The authors attributed such a behavior to the change of the crystallization process of PCL and/or to the poor interfacial adhesion between the nanohydroxyapatite particles and the PCL. In vitro biological tests results revealed the higher potential of the PCL/nanohydroxyapatite scaffolds to promote cell adhesion, proliferation, and osteogenic differentiation when compared to PCL/microhydroxyapatite scaffolds. Such a behavior was due to the formation of microhydroxyapatite particle aggregates on the surface of the scaffold, which prevents the establishment of cell-cell interactions essential for the biological processes. Therefore, the amount of nano-/microparticles embedded in a polymer matrix must be properly optimized to avoid weakness in the structure and to enhance cellular activity of composite scaffolds.

This manuscript addresses the development of biocomposite-based scaffolds towards characteristics suitable to tissue engineering. Preliminary tests with biocompatible materials were conducted for the evaluation of the adequate porosity and simultaneous capacity for ensuring cell growth and adequate mechanical strength compatible with the host tissue. Scaffolds of poly(*ε*-caprolactone) (PCL), as the polymeric matrix, with different amounts of Biosilicate® microparticles, as bioactive and ceramic reinforcement, were fabricated. Their morphological analysis, mechanical and biological properties, and influence of Biosilicate® on both properties of the polymer matrix and process variations of the additive technique by extrusion were evaluated.

## 2. Materials and Methods

### 2.1. Materials

Powder PCL CAPA® 6500, purchased from Perstorp®, has particle size smaller than 500 *μ*m, 50,000 g/mol average molecular weight (Mw), 60 to 65°C melting temperature, and approximately −60°C glass transition temperature. Biosilicate® of 23.75Na_2_O-23.75CaO-48.5SiO_2_-4P_2_O_5_wt.% composition, with one crystalline phase of sodium–calcium silicate (Na_2_CaSi_2_O_6_), average particle size (d_50_) between 180 and 212 *μ*m, and melting temperature above 1250°C [16, 20, 24], was supplied by Laboratory of Vitreous Materials at the Federal University of Sao Carlos.

### 2.2. Manufacture of Scaffolds

**Figure 1:**
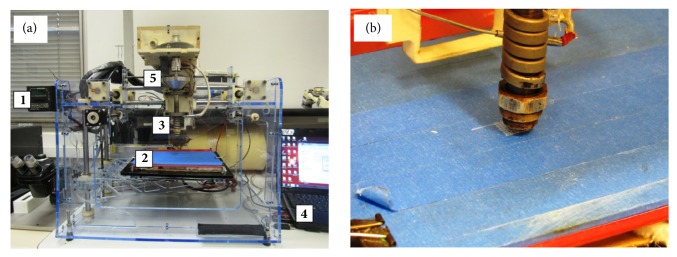
(a) Fab@CTI 3D printer, where 1: speed and temperature controller; 2: printing platform; 3: extrusion mini-screw head; 4: control computer; and 5: feed hopper. (b) Construction of the first layer under a 0°/90° deposition pattern.

Scaffolds of PCL with 0, 1, 3, and 5 wt.% of Biosilicate® biocomposites were fabricated by extrusion additive process in a Fab@CTI 3D printer (see Figure 1(a), where 3 indicates the extrusion miniscrew head composed of a single screw, barrel, and nozzle; 2 denotes the heated printing platform; 1 refers to the control system (rotating speed and temperature); 4 denotes the control computer; and 5 indicates the feed hopper). The PCL and Biosilicate® powders were weighed on an analytical balance and mechanically mixed prior to the addition to the feed hopper of the 3D printer.

Some operational parameters were defined in the preliminary tests for the establishment of a continuous and stable flow of materials through the 400 *μ*m inner diameter extrusion nozzle. A 7.5 rpm rotation speed and a 12 mm s^−1^ head deposition speed were adjusted for the manufacture of the scaffold compositions. The processing temperature was set to 67 to 96°C range, according to the preliminary tests.

Scaffolds of square geometry and 0°/90° deposition trajectory were printed, as shown in Figure 1(b). Geometric parameters were initially established for the fabrication of the scaffolds and correspond to their physical and structural properties, as layer height (PH), spacing between extruded filaments and perimeter, number of layers, and diameter of the desired extruded filament. All characteristics determined for the structure of the scaffold should consider the specifications and standards related to the desired application. A 0.30 mm layer height and a 0.9 mm spacing between the extruded strands were established and resulted in an approximately 0.45 mm pore size.

### 2.3. Morphological Characterization

The morphology of the scaffolds was analyzed under a FEI® Inspect F-50 scanning electron microscope (SEM) operating at 10 kV. Samples of 5 mm length, 5 mm width and 5 mm thickness were cut from the printed scaffolds by a scalpel and recovered with a thin layer of platinum by Q150R ES Sputter® Quorum Techonologies® equipment. Subsequently, the SEM images were assessed by ImageJ® version 1.60 software. The mean extruded strand diameter and mean pore size were determined through 100 random measurements.

### 2.4. Mechanical Characterization

The mechanical properties of the 3D printed scaffolds of 5 mm length, 5 mm width and 10 mm thickness were evaluated by mechanical compression tests, according to ASTM D2990–0990, in a model 5969 Instron® universal testing machine with a 1.5 kN load cell and operating at 1.3 mm/min test speed. The apparent compressive modulus was determined based on the slope of the linear region of the stress-strain curve.

### 2.5. Assessment of Cell Viability

The scaffold effects on cell viability were evaluated by* in vitro* assays with pre-osteoblasts MC3T3-E1 cell line (derived from rat calvarium) maintained in MEM Eagle medium (Gibco®, Life Technologies) supplemented with 10% fetal bovine serum, 1% penicillin-streptomycin (100 mg/mL, Vitrocell®), nucleosides and deoxynucleosides (9.9 mg/L adenosine, 10.8 mg/L deoxyadenosine; 10 mg/L cytidine, 10.4 mg/L deoxycytidine, 9.9 mg/L guanosine, 10.6 mg/L deoxyguanosine, 9.9 mg/L thymidine, and 10 mg/L uridine) in atmosphere supplemented with 5% CO_2_ at 37°C. The cell medium was changed every two days. Two layers of each scaffold were cut by a blade. The specimens were washed with ethanol (70% purity) and placed inside a cell culture hood for 1 h hour to dry and sterilized under UV light for 20 min. They were then placed in a 24-well tissue culture polystyrene (TCPS) plate and covered by a TCPS insert for avoiding floating. The cell viability was evaluated by the direct contact of the samples and the osteoblasts. MC3T3-E1 cells were trypsinized, counted, and seeded at 1 × 10^4^ cells per well. Towards keeping most of the cells in contact with the scaffolds, firstly the required number of cells per well in 100 *μ*L of cell culture media was suspended and the pores of the scaffolds were filled with such 100 *μ*L of cell suspension. Empty TCPS wells (with no sample) seeded with 100 *μ*L cell suspension were employed for control. The cells were allowed to adhere to either the scaffolds, or TCPS for 45 min at 37°C with 5% CO_2_. Each testing well was supplied with 1 ml cell culture media. Four wells/specimens were employed for each sample and the control. Resazurin staining (AlamarBlue®, Life Technologies™) was used for quantification, since it measures the cell viability by fluorescence or colorimetry. As it is oxidized when it passes through the viable cell mitochondria, a continuous cell growth produces an oxidized environment, which turns resazurin into fluorescent and red compounds. Therefore, no cell growth, or inhibition of cell growth keeps resazurin non-fluorescent and blue [32]. The cell viability was measured after 1, 3, 7, 14 and 21 days of cell seeding, which is considered a long term* in vitro* testing [32, 33]. The cell culture media were aspired for each measurement and 600 *μ*L of resazurin diluted in the culture media at 10 v% were added per well. Two empty wells (with no cells) were used as control of resazurin. The plates were kept at 37°C with 5% CO_2_ and, after 4 h, 100 *μ*L of the supernatant of each well, including the controls of resazurin, were transferred to a 96 well TCPS plate. Two wells with 100 *μ*L of full-reduced resazurin each, obtained through dilution at 10% (v/v) in the culture media and autoclaving of the solution at 120°C for 15 min, were employed as reference. Fluorescence (544 nm excitation, 590 nm emission wavelengths) was measured by a Spectra Max Gemini XS Molecular Devices instrument. The cell viability was estimated according to the relative reduction obtained for each period, calculated by

(1) $$\begin{array}{c} Relative\;\;fluorescence\left(\;\%\;\right)=\frac{sample\;\;fluorescence-resazurin\;\;control\;\;fluorescence}{100\%\;\;reduced\;\;resazurin\;\;fluorescence-resazurin\;\;control\;\;fluorescence}\times 100 \end{array}$$

Statistical comparisons were performed by two-way ANOVA on GraphPad Prism® software and Bonferroni multiple comparisons test. P values < 0.05 were considered statistically significant (n=4).

## 3. Results and Discussion

### 3.1. Morphological Characterization

**Figure 2:**
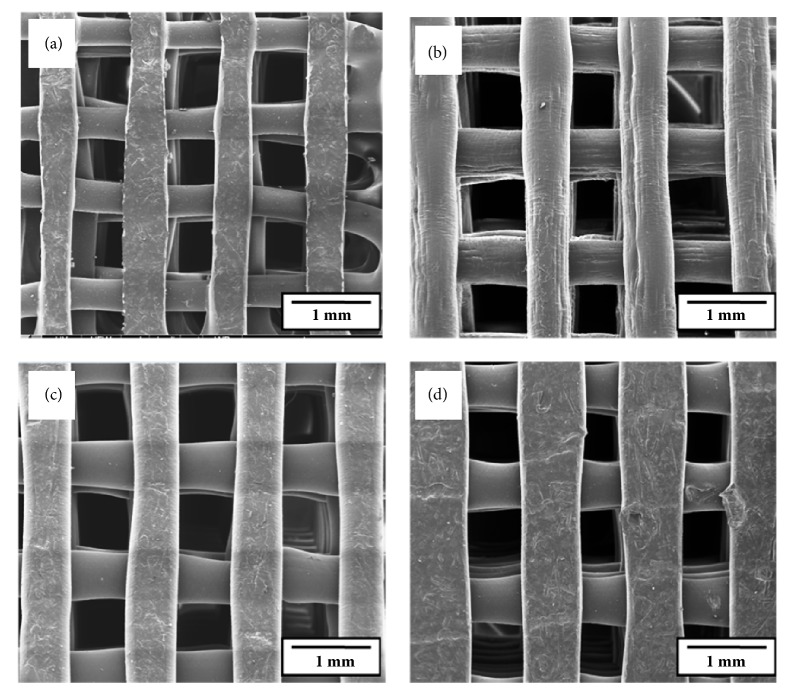
SEM images of the XY-plane top views of the fabricated scaffolds: (a) PCL; (b) PCL + 1 wt.% Biosilicate®; (c) PCL + 3 wt.% Biosilicate®; and (d) PCL + 5 wt.% Biosilicate®.

Initially, the print quality of Fab@CTI was evaluated.  Figure 2 shows SEM micrographs of the XY-plane top views of the fabricated scaffolds with different compositions. The 0°/90° architecture proposed for the scaffolds was obtained, which confirmed the compatibility between BioScaffold PG geometry generation software and 3D printer linked program, Fab@Home. Such an architecture is known to guarantee interconnectivity between pores, which is a determining geometric factor for the application of scaffolds in tissue engineering [11, 31]. The reproducibility factor, which consists in the manufacture of scaffolds with similar compliance, is observed mainly in Figures 2(a), 2(b), and 2(c) and related to the scaffolds with Biosilicate®. The structures are homogeneous and uniform and exhibit a similar pore and extruded strand geometry. The scaffolds fabricated with pure PCL showed lower compliance in comparison to scaffolds with bioceramic particles (Figure 2(d)).

**Figure 3:**
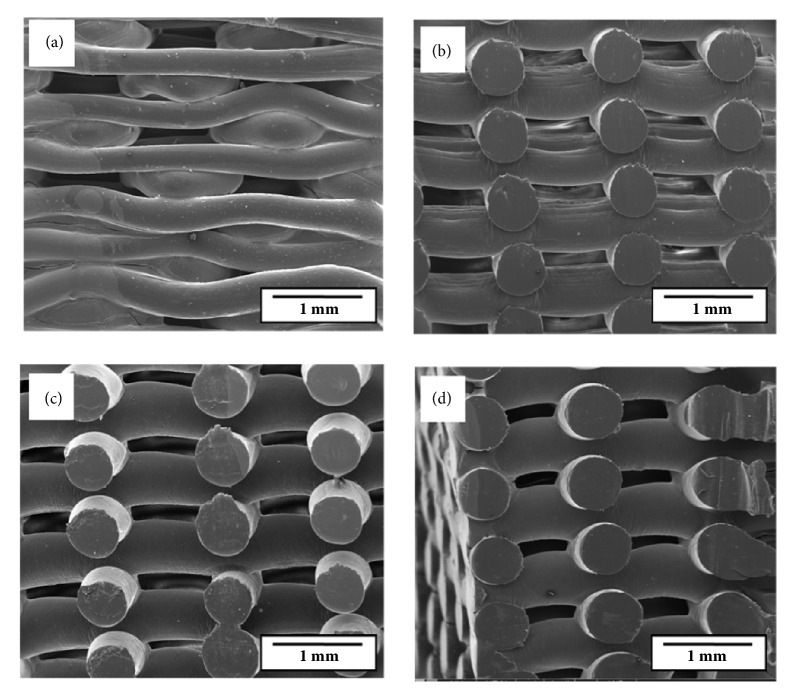
SEM images of the fabricated scaffolds: (a) lateral surface of PCL; and cross sections of (b) PCL + 1 wt.% Biosilicate®; (c) PCL + 3 wt.% Biosilicate®; (d) PCL + 5 wt.% Biosilicate®.

The bonding between the layers of the scaffolds was evaluated by the SEM micrographs shown in Figure 3. The analysis investigated the quality of the layer height (PH) parameter, considering bonding, homogeneous distribution and size (filaments). As known, the higher the PH, the poor the layers bonding, therefore, an inadequate definition of the layer height can result in the detachment of the layers from the structure. On the other hand, the presence of flattening in the regions of union of one layer to another, caused by the lower PH in relation to the diameter of the extruded strand, is indicative of good bonding [31]. Figures 3(b)–3(d) show SEM images of the cross sections of the fabricated scaffolds of PCL with 1, 3, and 5 wt.% Biosilicate®, respectively. The bonding between the layers increased as the bulk percentage of the bioceramic material was increased, due to the gradual increase in the diameter of the extruded strand in relation to the fixed layer height (PH = 0.30 mm), as the mass of Biosilicate® in the composition increased. Figure 3(a) shows the SEM micrograph of the lateral surface of the scaffold structure fabricated from pure PCL. The PCL scaffolds showed the poorest bonding between layers in comparison to the scaffolds of other compositions. Such a behavior was perceptible even during the handling of the samples. The pore cover observed in lower layers is caused by the decay of the extruded strands in the deposition process.

**Figure 4:**
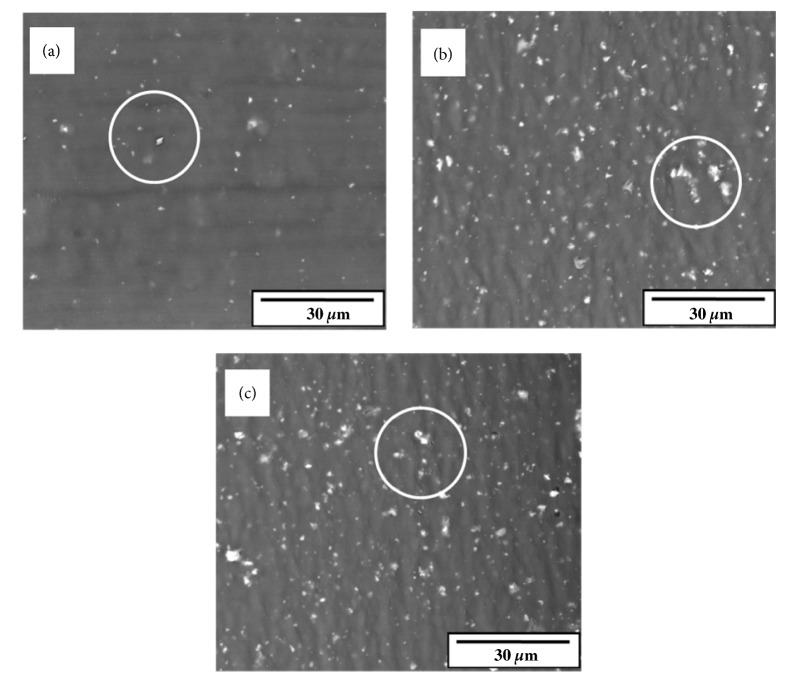
SEM images of scaffold surfaces: (a) PCL + 1 wt.% Biosilicate®; (b) PCL + 3 wt.% Biosilicate®; (c) PCL + 5 wt.% Biosilicate®. Circles indicate some agglomerates.

Figures 4(a), 4(b), and 4(c) show higher magnification SEM micrographs of the surface of scaffolds with 1, 3, and 5 wt.% Biosilicate®, respectively. As expected, the number of Biosilicate® particles increased on the surface of the scaffold, as their concentration in the biocomposite increased. All samples showed a good distribution of the bioceramic particles; however, a low dispersion of the particles due to the presence of some agglomerates. The presence of agglomerates is probably due to the limited mixing power of the extruder screw and the nozzle clogging during printing. The mixing power of the extruder screw will be subject of further studies. Dávila et al. [11] and Domingos et al. [27] also reported the formation of aggregates, leading to a rough and heterogeneous distribution of the bioceramic particles within the scaffolds.

The number of bioceramic particles added to the polymer matrix can interfere with the flow of the material in the manufacturing process, altering the resulting morphological characteristics.  Table 1 shows the results of average strand diameter and average pore size of the scaffolds. The diameter of the extruded strands increased, as the amount of Biosilicate® in the biocomposite increased. Such a behavior is due to the extruded swell phenomenon, which occurs in the extrusion process of the material through the die of the 3D printer, and factors, as speed and rotation of the screw. The increase in the diameter of the extruded strands is the result of the elastic recovery of the polymer, as observed by other authors [11]. Kyriakidou* et al*. [34] reported the influence of deposition speed on the diameter of the extruded filament of three-dimensional PCL/hydroxyapatite cylindrical scaffolds. The scaffolds obtained at a high speed showed a porous structure compromised due to strand–strand fusion within the same layer. At a low speed, the bonding between adjacent layers was limited and compromised the structural integrity of the scaffold. Evidently, the strand diameter plays an important role in the determination of the pore size of the scaffold. Nevertheless, it is further influenced by other parameters, as layer height and spacing between the extruded strands.

The existence of well-defined and interconnected pores is fundamental for cell adhesion and migration efficiency. Furthermore it is well known that the pore structure is a vital factor that can significantly affect the mechanical properties of scaffolds. Therefore, another aspect to be analyzed is the pore size resulting from the manufacture of the scaffolds. A comparative analysis was also the object of this study. The results (Table 1) show the average pore sizes of the scaffolds decreased, as the amount of Biosilicate® in the biocomposite increased. Additionally the pore sizes of all manufactured scaffolds were within the range of tissue engineering applicability, i.e., 50 to 750 *μ*m, as described in the literature [11, 35]. As expected, the scaffolds of larger strand diameters showed smaller pore sizes, since they were printed maintained the structure and the printing parameters.

### 3.2. Mechanical Characterization

**Figure 5:**
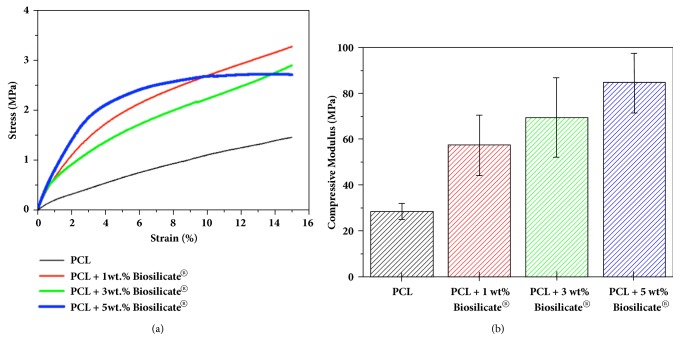
(a) Average stress-strain curves and (b) compressive modulus of PCL, PCL + 1, 3, and 5 wt.% Biosilicate® scaffolds.

The mechanical properties of the scaffolds must be in accordance with the demands required for the host tissue and their structure must enable tissue regeneration. Figures 5(a) and 5(b) show the average stress-strain curves of the fabricated scaffolds and the results of compressive modulus and standard deviation of the measurements, respectively. The pure PCL scaffolds exhibited a compression modulus of 28.2 ± 3.5 MPa with a smaller variation. The value of compressive modulus is lower in comparison with the values observed by other authors [5, 11, 36] and can be explained by the lack of bonding between the layers and the lower diameter and pore size conformity of the extruded strands of the pure PCL scaffolds, which resulted in structures with lower mechanical characteristics. The stress-strain curves of the scaffolds of PCL with Biosilicate® exhibited an elastic behavior in the linear region at low stress values, followed by a long plateau as the deformation increased (see Figure 5(a)), as reported by other authors [11, 34].

The results in Figure 5(b) show an increase in the compressive modulus of the polymer matrix in function of the increase in the amount of Biosilicate®. The specific scaffold stiffness increases approximately 22% when the bulk percentage of Biosilicate® increases from 1 to 3 wt.%, while the specific stiffness increase was approximately 57% when the Biosilicate® content increased from 1 to 5 wt.%. The compressive modulus of PCL / Biosilicate® scaffolds ranged between 58.2 ± 12.2 MPa and 85.4 ± 13.1 MPa. Therefore, all scaffold compositions studied, including PCL with no Biosilicate®, met the required specifications regarding rigidity for bone regeneration applications. According to Goonoo et al. [37] and Yang et al. [38], 20 to 141 MPa is the ideal stiffness range for the application of scaffolds in bone tissues. The greater variation in the compressive moduli in all PCL / Biosilicate® scaffolds is probably due to the partial agglomeration of Biosilicate®, as revealed by the SEM analysis, Figure 4.

Nevertheless, it should be highlight that the mechanical properties of the scaffolds are significantly influenced by the pore structure, and not only by the composition of the material.

In the case of the fabricated PCL / Biosilicate® scaffolds, the higher compressive modulus was observed for the scaffold with smaller pore sizes, i.e., PCL + 5 wt.% Biosilicate® scaffold.

### 3.3. Assessment of Cell Viability

**Figure 6:**
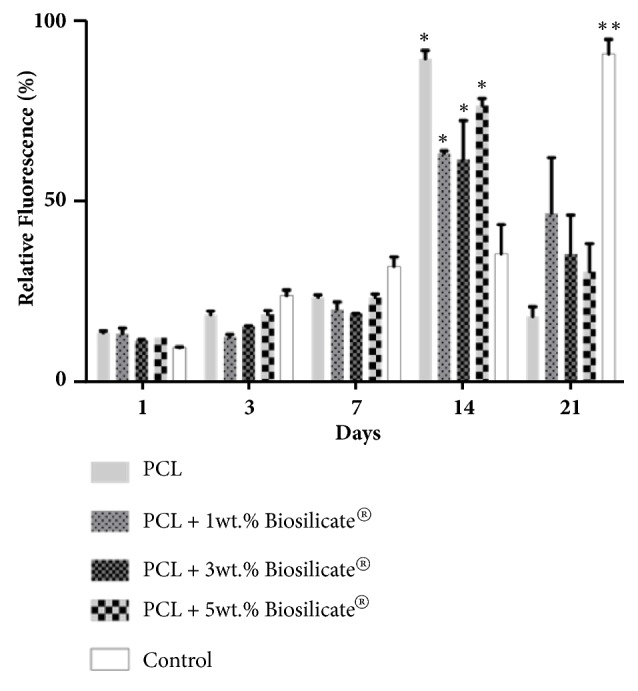
Cell viability of the scaffolds. Each column represents the mean ± SEM (Standard Mean Error) between the samples. *∗* n < 0.05 for the scaffolds in comparison with the control; *∗∗* n < 0.05 for the control in comparison with all scaffolds.

Figure 6 shows the cell viability of PCL + Biosilicate® composites in direct contact with the pre-osteoblasts MC3T3-E1 cell line, measured by resazurin. The MC3T3-E1 cells proliferated until day 14 for all samples, which indicates the scaffolds show no-toxicity* in vitro* and support cells proliferation. A stable relative fluorescence was observed for all samples until day 7, therefore, no significant differences were detected between the samples and the control. A significant higher cell viability rate was observed for all scaffolds in comparison with the control (cells with no scaffolds) on day 14. The highest fluorescence values were obtained for pure PCL scaffolds, with significant differences in comparison with PCL + 1 wt.% Biosilicate® and PCL + 3 wt.% Biosilicate®. Such changes could not be detected in the early days of the assays, because a relatively low cell number (1 × 10^4^) was used for keeping the cell culture stable until the end of the assays (21 days).

Since those differences are not constant in the samples, i.e., the cell viability rates do not change significantly between the neat PCL scaffolds and PCL + 5 wt.% Biosilicate®, the lower proliferation rate cannot be attributed to Biosilicate®. Although most Biosilicate® is embedded within the PCL matrix, the bioceramic particles can be visualized on the surface of the PCL scaffolds through SEM images (Figure 4). Biosilicate® is a bioactive glass that releases ions (Ca^+^, P^+^ and Si^+^) and, thus, increases pH. Such changes can affect the cell proliferation in the early stages of* in vitro* studies, even if the cell medium changes every two days, as reported by Montazerian* et al*. [39] and Xynos* et al*. [40], who investigated osteoblasts proliferation of bioactive glasses. However, according to Xynos* et al*. [41] and Valerio* et al*. [42], Ca^+^, P^+^ and Si^+^ exert beneficial effects on osteoblasts proliferation. Under* in vivo* conditions, the pH shift may be absent, since the body pH is controlled by buffer systems [43]. An increase in the number of particles also increases the roughness of the scaffolds surface, which is beneficial for cell attachment and proliferation [44, 45]. Therefore, the higher roughness of the scaffolds with 5 wt.% Biosilicate® may compensate for the cell proliferation.

After 21 days of assays, the cell viability decreased for all scaffolds. Under* in vitro* conditions, the cells proliferate until all available surfaces have been covered and no space is available and, finally, the apoptosis cascade is signalized. Apoptosis, the process of cell death, is fundamental under physiological conditions and critical for maintaining the normal development and function of multicellular organisms [46, 47]. Therefore, if the* in vitro* cell proliferation quickly reaches a high value, the proliferation also decreases fast, because of the apoptosis signaling in function of space saturation. Although PCL scaffolds provided the highest osteoblasts proliferation rates after 14 days of cell seeding, they also showed the highest cell decrease at 21 days of assay. At this period, the cells proliferation in samples with Biosilicate® was more continuous, because the Ca^+^ ion release is beneficial for osteoblast proliferation; it increases the gene expression of essential osteoblast growth factors [41] and may compensate for the apoptosis signaling.

The control (cells on TCPS) showed a stable viability curve over time, because of the presence of fewer variables (polymer, pH, surface) in comparison with osteoblasts cultured within the scaffolds. However, the control provided lower proliferation rates in comparison with the scaffolds until day 14. This behavior corroborates with the hypothesis that 3D scaffolds improve the cell proliferation due to the higher surface area in comparison with the flat surface of the TCPS well. Under in vitro conditions, the cell proliferation is viable until the saturation of the surfaces [48–50].

Overall, the osteoblasts proliferated in all samples with no significant differences, except at day 14 for scaffolds and control, due to the larger surface of the scaffolds in comparison with the TCPS plate surface. The cell viability of the pure PCL sample was significantly higher in comparison with PCL + 1, 3 and 5 wt.% Biosilicate®, probably due to the ions release. The higher roughness of the scaffolds of PCL + 5 wt.% Biosilicate® sample additionally increased the cell adhesion and proliferation.

Further* in vitro* studies, as transcriptase Polymerase Chain Reaction (rt-PCR), are necessary for the understanding of the way the ion release from composite scaffolds can affect the cell proliferation. We propose* in vivo* studies as a clinical translation step for scaffolds, towards understanding the physiological dynamics of the scaffold-tissue interface in a long term, as well the biodegradation behavior.

## 4. Conclusions

The SEM morphological characterization of scaffolds produced by 3D printing obtained a desired 0°/90° architecture, which indicates the excellent compatibility between the software involved in the process and the 3D printer. The layer height (PH) values, which must be lower than the diameter of the extruded strand, must be considered for a better bonding between the scaffold layers. Therefore, scaffolds composed of PCL + 5 wt.% Biosilicate® promoted a superior bonding between the layers in comparison to the scaffolds of other compositions. The processing conditions used in the preparation of biocomposites in the mini-screw extruder induced a good distribution of Biosilicate® particles in the PCL matrix; however, some agglomerations of the bioceramic material were found on the surface of the scaffolds. The extruded swell phenomenon, which is directly related to the number of particles in the matrix, was identified. Therefore, the increase in the diameter of the extruded strand in relation to the inner diameter of the nozzle must be considered in the scaffold structure design. The pore sizes of the fabricated scaffolds are within the range of application to tissue engineering and the pores showed good interconnectivity. The results of mechanical compression tests revealed significant improvements in the mechanical properties of the scaffolds with the addition of Biosilicate® in the polymer matrix. Scaffolds with 3 and 5 wt.% bioceramic material showed 22 and 57% increases in stiffness, respectively, in relation to scaffolds with 1 wt.% Biosilicate®. All manufactured scaffolds met the specific stiffness specifications for applications in bone tissue engineering. Cell viability results showed cell adhesion and proliferation throughout the tests of fabricated scaffolds and the non-toxicity of scaffolds to MC3T3-E1 pre-osteoblast cells was evidenced. The results of morphological, mechanical and biological characterizations proved such scaffolds can be applied to tissue engineering for bone reconstruction.

## Figures and Tables

**Table 1:** Mean extruded strand diameter and mean pore size.

Sample	Strand diameter (*μ*m)	Pore size (*μ*m)
PCL	445.91 ± 44.83	655.82 ± 97.50
PCL + 1 wt.% Biosilicate®	579.81 ± 18.83	545.39 ± 41.88
PCL + 3 wt.% Biosilicate®	607.76 ± 30.55	475.34 ± 57.34
PCL + 5 wt.% Biosilicate®	735.17 ± 20.35	374.88 ± 41.39

## Data Availability

All data presented have been manually entered in datasets and are available from our first and corresponding authors for inspection upon request.
